# Connecting marine productivity to sea-spray *via* nanoscale biological processes: Phytoplankton Dance or Death Disco?

**DOI:** 10.1038/srep14883

**Published:** 2015-10-14

**Authors:** Colin O’Dowd, Darius Ceburnis, Jurgita Ovadnevaite, Jakub Bialek, Dagmar B. Stengel, Merry Zacharias, Udo Nitschke, Solene Connan, Matteo Rinaldi, Sandro Fuzzi, Stefano Decesari, Maria Cristina Facchini, Salvatore Marullo, Rosalia Santoleri, Antonio Dell’Anno, Cinzia Corinaldesi, Michael Tangherlini, Roberto Danovaro

**Affiliations:** 1 School of Physics & Centre for Climate and Air Pollution Studies, Ryan Institute, National University of Ireland Galway, University Road, Galway, Ireland; 2 School of Natural Sciences & Centre for Climate and Air Pollution Studies, Ryan Institute, National University of Ireland Galway, University Road, Galway, Ireland; 3 CNR-ISAC, Bologna, Italy; 4 Agenzia nazionale per le nuove tecnologie, l’energia e lo sviluppo economico sostenibile, ENEA — Centro Ricerche Frascati, Frascati, Italy; 5 CNR-ISAC, Rome, Italy; 6 Department of Life and Environmental Sciences Polytechnic University of Marche, Ancona, Italy

## Abstract

Bursting bubbles at the ocean-surface produce airborne salt-water spray-droplets, in turn, forming climate-cooling marine haze and cloud layers. The reflectance and ultimate cooling effect of these layers is determined by the spray’s water-uptake properties that are modified through entrainment of ocean-surface organic matter (*OM*) into the airborne droplets. We present new results illustrating a clear dependence of *OM* mass-fraction enrichment in sea spray (*OM*_*ss*_) on both phytoplankton-biomass, determined from Chlorophyll-a (*Chl-a*) and Net Primary Productivity (*NPP*). The correlation coefficient for *OM*_*ss*_ as a function of *Chl-a* increased form 0.67 on a daily timescale to 0.85 on a monthly timescale. An even stronger correlation was found as a function of *NPP*, increasing to 0.93 on a monthly timescale. We suggest the observed dependence is through the demise of the bloom, driven by nanoscale biological processes (such as viral infections), releasing large quantities of transferable *OM* comprising cell debris, exudates and other colloidal materials. This *OM*, through aggregation processes, leads to enrichment in sea-spray, thus demonstrating an important coupling between biologically-driven plankton bloom termination, marine productivity and sea-spray modification with potentially significant climate impacts.

The marine aerosol produces haze and cloud layers overlying an immense ocean covering >70% of the Earth’s surface. Small changes even in low-albedo layers superimposing this relatively dark surface can have profound effects on the global radiation budget and climate change. Organic matter mass-fraction enrichment in sea spray aerosol (*OM*_*ss*_, defined here as the percentage *OM* mass in sea spray relative to the total *OM plus* sea salt mass) influences the global albedo through altering the reflectance of marine haze^1^ and cloud layers^2^. Recent results^3^ assert that a relatively constant sea surface carbon pool controls *OM*_*ss*_ rather than biomass abundance or biological productivity and that the constant pool leads to a constant sub-micron sea spray aerosol *OM*_*ss*_ of the order of 10%. *OM* in marine aerosol significantly alters the albedo of these layers due to its influence on hygroscopic properties^4^. Recent studies lead to contrasting perspectives on the relative contribution of water-soluble *OM* (*WSOM*) and water-insoluble *OM* (*WIOM*) to *OM*_*ss*_ in primary-produced sea spray aerosol^5^ from bubble-bursting process and its ultimate cloud droplet nucleating properties^6^,^7^.

Bubble-mediated spray production studies in a ship borne laboratory cruising the northeast Atlantic during a phytoplankton bloom, revealed *WIOM* enrichment up to 80% in submicron sea spray aerosol mass^8^ while other studies using laboratory-cultures of microalgae^7^ report enrichments of less than 30%. Analysis of enrichment fractions as a function of biological activity, using changes in chlorophyll-a (*Chl-a*) concentration as a surrogate of surface photosynthetic primary production, also lead to conflicting results. Satellite measurements of *Chl-a*^9^,^10^ yielded a significant correlation between enrichment and *Chl-a*, and hence biological activity, leading to enrichment parameterisations being developed for global models; however, other studies^3^ relating instantaneous *Chl-a* to *OM*_*ss*_ found no relationship, reporting that the organic carbon content of freshly emitted sea spray aerosol was effectively invariant at 5%, despite significant variability in seawater *Chl-a* levels. The former studies were conducted over the northeast Atlantic while the latter were conducted in the northwest Atlantic seas to the southwest of Massachusetts and the Pacific California. In the latter study, they^3^ found that the chemical composition of sea spray organics remained invariant from low-to-high chlorophyll conditions. Based on the above results, it was concluded that oceanic primary organic aerosol production is regulated by the static Dissolved Organic Carbon (*DOC*) reservoir and consequently, *OM*_*ss*_ and cloud nucleation activity of nascent sea spray aerosol is relatively constant over the global ocean.

## Results

We conducted two types of experiments: one producing a multi-year dataset spanning the period from 1^st^ January 2009 till 30^th^ September 2011 and covering three phytoplankton bloom periods while operating a continuous measurement programme of *OM*, sea salt and *OM*_*ss*_ in conjunction with a daily-timescale reanalysis dataset of satellite-derived biological proxy outputs (i.e. *Chl-a* and Net Primary Productivity - *NPP*, – see methods section); and a second type comprising discrete bubble-bursting experiments in laboratory tanks using water cultured in the *E. huxleyi* phytoplankton species, one of the most abundant species in the NE Atlantic. Both experiments where undertaken at the Mace Head atmospheric research station^11^, a northeast Atlantic World Meteorological Organisation Global Atmospheric Watch station which allowed the unique opportunity to gather the single-most extensive dataset on *OM*_*ss*_ enrichment in sea spray aerosol, carried in air advecting from highly productive oceanic waters, using state-of-the-art aerosol mass spectrometry. *OM* and sea salt concentrations were derived from High Resolution Time of Flight Aerosol Mass Spectrometry (*AMS*) measurements. Standard measurement and calibration procedures^12^,^13^ were applied for the *OM* measurements, with the modified analysis to derive sea salt concentration^14^. Sea spray enrichment factor was calculated as a ratio of the organic matter and the total sea spray. For the bubble-mediated mesocosm experiments, phytoplankton species were cultured in low-bacteria natural sea water utilising bubble-mediated spray production tanks similar to those used in previous ship-borne experiments amidst plankton blooms^8^.

**Figure 1:**
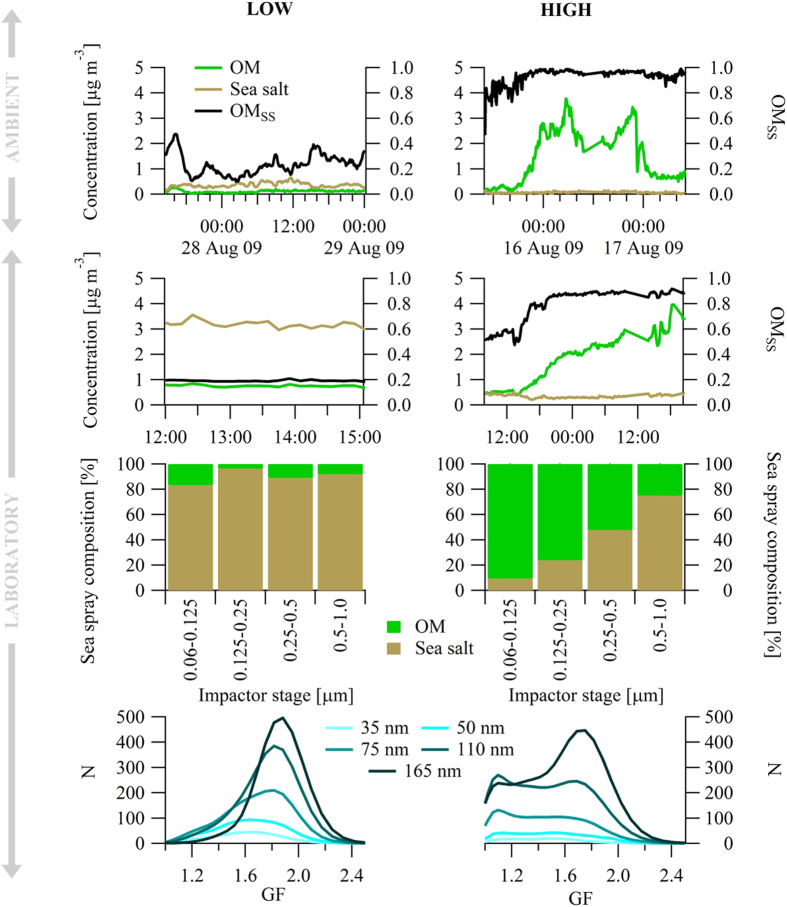
Left column and Right column plots relate to low *OM*_*ss*_ experiments and high *OM*_*ss*_ ‘explosions’, respectively. **(Top row)** OM and sea salt mass concentration and *OM*_*ss*_ for ambient air events. These events are 10 days apart for marine air during August 2009 under conditions of similar biological activity, using *Chl-a* as an indicator, and sea spray production as quantified by wind speed: for the low enrichment event, *Chl-a* was 0.82 μg L^−1^ and wind speed was 14 m s^−1^ while for the high enrichment case, *Chl-a* was 0.95 μg L^−1^ and wind speed was 11 m s^−1^. **(2**^**nd**^ **Row)** *OM* and sea salt mass concentration and *OM*_*ss*_ for mesocosm experiments. For the low enrichment mesocosm experiment, cell density, *Chl-a*, *DOC* and *POC* were 10^8 ^mL^−1^, 20.6 μg L^−1^, 111 μM, and 332 μM, respectively while for the high enrichment mesocosm experiment, cell density, *Chl-a*, *DOC* and *POC* were 10^5^ cells mL^−1^, 5 μg L^−1^, 140 μM, and 21 μM, respectively. **(3rd Row)** Percentage *OM* and sea salt as a function of spray particle size sampled by the Berner Impactor during the mesocosm experiments. **(Bottom Row)** Hygroscopic Growth Factor (*GF*) frequency distribution for mesocosm experiments. *GF* is the increase in particle size as the carrier air relative humidity (RH) is increased from <10% *RH* to 90% *RH*. For pure sea salt, the *GF* is 2.2 while *GF* for pure *POM* is 1.1. The figure illustrates that for the low enrichment experiment, the particles are predominantly sea salt with a smaller fraction of *OM* leading to a *GF* slightly lower than pure sea salt (i.e. 1.85 for the larger sized particles and 1.6 for the smallest). For the high enrichment experiment, where the percentage mass contribution increases with particle size, a more dominant very hydrophobic mode at *GF *= 1.1 becomes more prominent.

In both ambient air experiments and bubble-tank experiments, we observed the occurrence of striking *OM* ‘enrichment bursts’ (Fig. 1). During these bursts, the absolute ambient submicron *OM* mass concentration increased 10-fold and exceeded ~4 μg m^−3^ while *OM*_*ss*_ exceeded 95%. Similarly, in the bubble-tank culture experiments, during the *OM* bursts, *OM*_*ss*_ exceeded 95% and mass concentrations, scaled to ambient air particle number concentrations, also approached an equivalent concentration of 4 μg m^−3^ (actual concentrations were more than 20 μg m^−3^). For the ambient enrichment bursts, environmental and biological proxy data were comparable with marginally higher *Chl-a* concentrations and marginally-lower wind speeds (see Figure legend) while in the mesocosm experiments, phytoplankton abundance, *Chl-a*, Dissolved Organic Matter (*DOM*) and Particulate Organic Matter (*POM*) were all significantly higher in the non-explosion case.

Also shown is the chemical composition (*OM* and sea salt) measured in four size ranges sampled using a Cascade Impactor^15^ covering 60 nm to 1 μm size and the hygroscopic growth factor (*GF*-see methods) for 35 nm, 50 nm, 75 nm, 110 nm and 165 nm particles for the two selected bubble-tank experiments. In the impactor data, without the enrichment explosion, *OM*_*ss*_ is between 3% and 18% over the four stages whereas, for the enrichment explosion case, the enrichment increases from 25% at the largest sizes to more than 90% at the smallest sizes. A similar pattern in hygroscopic growth is seen with a monomodal frequency distribution of *GF* centred on 1.85 (very hygroscopic) reflecting an internal mix of predominantly sea salt and a minority of *OM* matter for the case of low enrichment and for high enrichment, the profound shift in *GF* to 1.1 (i.e. barely hygroscopic) as particle size reduces and enrichment increases are seen.

The significance of these experiments is that they demonstrate, for the first time, that the *OM*_*ss*_ bursts occur both in ambient air experiments and laboratory experiments and not only do they reaffirm that *OM*_*ss*_ contributions can reach 95% submicron mass while simultaneously leading to ambient air *OM* mass contributions 10-fold higher than other studies reported, but they also show approximately an order of magnitude variability in *OM*_*ss*_ , again in stark contrast to the Quinn *et al.*^3^ study. Perhaps even more striking is that the bursts occur in a pattern independent of instantaneous productivity markers and wind-generated forcing, both for the laboratory and ambient air experiments. While the lack of correlation between *OM*_*ss*_ and instantaneous productivity markers is in agreement with the findings of Quinn *et al.*^3^, both the large absolute *OM* concentration, variation in *OM*_*ss*_, and magnitude of *OM*_*ss*_ is in stark contrast to their results.

**Figure 2:**
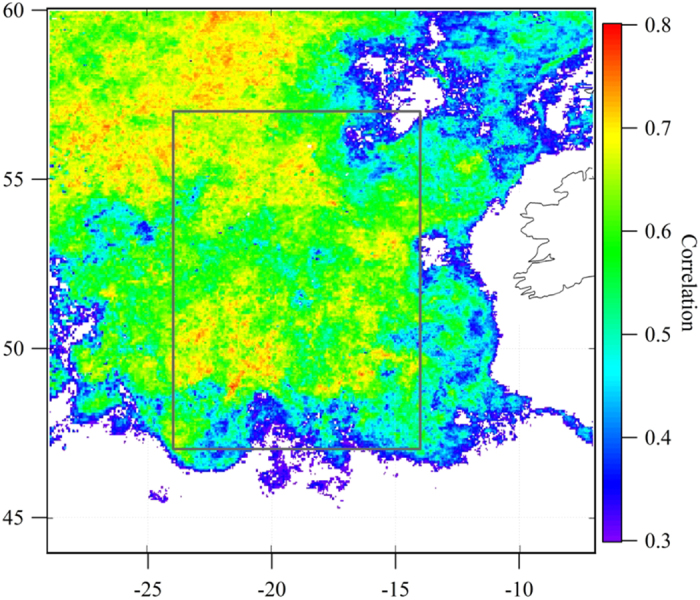
The correlation map of sea spray enrichment, measured at Mace Head, and *Chl-a* over the North East Atlantic. Pixel size is 0.042° (~5 km). Correlation was calculated from daily mass-fraction enrichment and *Chl-a* values for the period of 2009–2011. Only clean maritime polar air masses were taken into account. The 8 day lag between the enrichment and *Chl-a* was used in this analysis. Grey box represents an area of 47°N-57°N and 14°W-24°W^10^. The map was created by IGOR Pro 6.

To explore further any correlation between enrichment, phytoplankton biomass (as *Chl-a* concentrations) and productivity (as *NPP*), the multi-year dataset was analysed for a selected region (between 47°N- 57°N and 14°W-24°W, see Fig. 2) upwind of Mace Head. The cleanest air mass with least continental and/or anthropogenic contamination encountered in the region is a maritime polar air mass, therefore, only these air masses were selected for the sea spray composition analysis. An additional black carbon criterion of 1 hour average mass concentration not exceeding 15 ng m^−3^ was applied to the data in order to ensure the cleanliness of the marine air masses. *Chl-a* averages over the selected region were filtered for the same periods that corresponded to these clean air criteria. The air mass characterisation was conducted on a 6 hour time resolution and, consequently, the enrichment factor was averaged over the 1 hour period around the air mass classification. Since the highest *Chl-a* time resolution was 24 hours, its concentration was assumed to be constant for that particular day. Also shown in the Fig. 2 is the correlation coefficient between *Chl-a* and *OM*_*ss*_ with an 8-day time lag, illustrating the regions of biomass abundance strongly correlated with enrichment measured at Mace Head. An 8-day time-lag was previously found^10^ to lead to a maximum correlation between *Chl-a* and *OM*_*ss*_ and was regarded^10^ as a biological time-lag between peak biomass abundance and peak *OM*_*ss*_ in spray.

**Figure 3:**
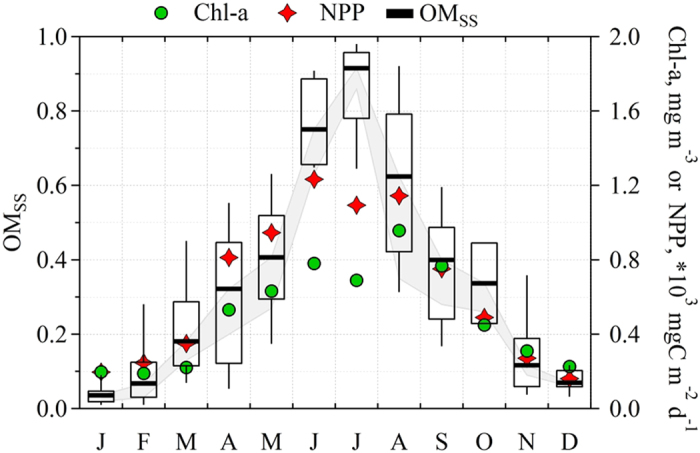
Temporal seasonality of *OM*_*ss*_ in sea spray aerosol, sea water chlorophyll-a (*Chl-a*) and Net Primary Production (*NPP*). *OM*_*ss*_ bold lines represent median concentrations, boxes- 25–75% percentile and whiskers demonstrate 10–90% percentile. Grey area represents a reduction in primary *OM*_*ss*_ due to possible secondary *OM* contribution to total organics. The secondary *OM* contribution was derived from data presented in Ceburnis *et al.*^55^ and ranges from 22% to 74% depending on the month.

The magnitude and variation of *OM*_*ss*_ is also clearly seen in the analysis of the 3 year dataset as displayed in Fig. 3 where *OM*_*ss*_ values increase from 4% in winter to greater than 90% in summer as *Chl-a* increased from 0.2 mg m^−3^ to 0.96 mg m^−3^ and *NPP* from 161 mgC m^−2^ d^−1^ to 1234 mgC m^−2^ d^−1^. Of course, the *OM* fraction derived from *AMS* measurements would include secondary *OM* contribution as well. So far, there is no reliable method to routinely separate primary and secondary marine aerosol *OM*, since they could bear the same properties, especially, after aging^16^. Nevertheless, even high secondary contribution would not change the whole picture very much. By way of illustrations, during the warm, sunny, period of summer, where high secondary contribution could be expected, an extraordinly high 70% classification of *OM* as secondary organic aerosol would result in only ~15% reduction in *OM*_*SS*_, as high *OM*_*SS*_ in such periods is determined by contextually-high *OM* and low sea salt concentrations. *OM* variations could have larger impact on low *OM*_*SS*_ values occurring during the winter period. Nevertheless, the contribution from secondary *OM* during winter is expected to be minumum, resulting eventually in insignificant effect on *OM*_*ss*_. In other words, secondary contribution could scale down the *OM*_*ss*_ by no more than 15% over all seasons, leaving the observed yearly trend unchanged. This is illustrated in Fig. 3.

**Figure 4:**
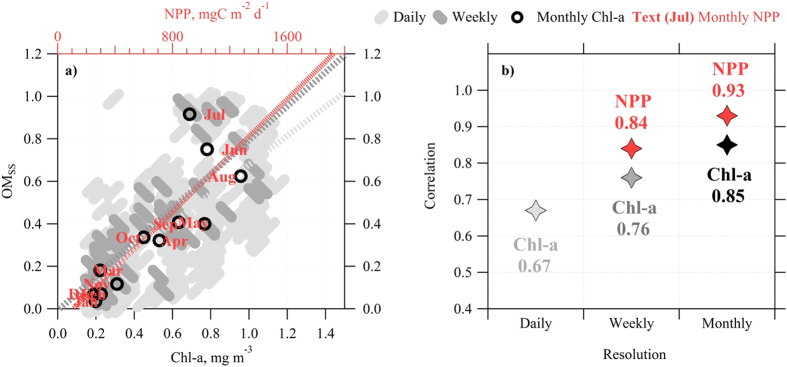
(**a**) Scatter plot of sea spray *OM*_*ss*_ as a function of *Chl-a* for daily, weekly and monthly temporal resolutions and *NPP* for monthly temporal resolution; (**b**) correlation coefficient as a function of temporal resolution of biological indicators.

The shortest timescale analysed using the satellite data was one day and led to a correlation coefficient between *Chl-a* and *OM*_*ss*_ of *R *= 0.67 (Fig. 4) while no correlation in instantaneous timescales was observed from the bubble-tank experiments. Moreover, Fig. 4 illustrates that as the timescale was lengthened to one week, the correlation increased to 0.76, and up to 0.85 for a month-long timescale while *NPP* exhibited a correlation of 0.93, again for a month–long timescale, confirming that *OMss* is neither constant nor independent of biological activity.

The aforementioned correlation maximum for monthly average concentrations, combined with the observations of a 26-day “boom-bust” biological cycle timescale associated with bloom dynamics as recently illustrated by Lehahn *et al.*^17^, suggests that the maximum correlation occurs at a timescale longer than 8 days and perhaps the peak in *OM*_*ss*_ is more associated with the demise of the bloom rather than any growth phase.

**Figure 5:**
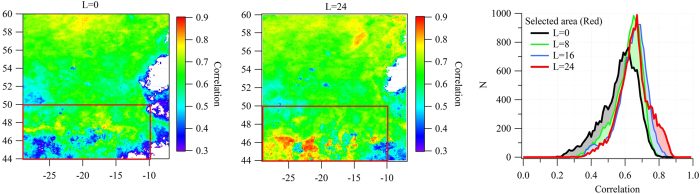
Correlation coefficient spatial distribution between *OM*_*ss*_ and *NPP* for 0 days time lag (left) and for 24 days time lag (middle) at each grid cell upwind of Mace Head. The correlation coefficient frequency distribution is also shown (right) for area comprising grid coordinates 44° and 50° N and −10° and −30°W (the area overwhich trajectories advect during the strongest *OM* plumes) for time lags of 0, 8, 16, and 24 days. The maps were created by IGOR Pro 6.

This suggestion is supported here through examination of the correlation coefficient frequency function for *NPP*, taken over the grid region from 44° and 50° N and −10° and −29°W (the area overwhich trajectories advect during the strongest *OM* plumes) for time lags of 0, 8, 16, and 24 days. Figure 5 illustrates the correlation between *NPP* and *OM*_*ss*_ over the chosen grid and illustrates that the correlation coefficient distribution function both narrows and increases in both amplitude and peak distribution value as the time lag is increased from 0 to 16–24 days. This suggests a maximum correlation between *OM*_*ss*_ and *NPP* for the latter third of a typical bloom life span, or, in other words, maximum correlation is seen for bloom demise rather than bloom decay. The width of the distribution function, and the lack of a mean correlation as high as that seen for averages over weekly and monthly timescales shown in Figs 3 and 4 is most likely due to blooms, typically active at the meso-scale of approximately 200 km in size^16^, waxing and waning out of synchronization with each other.

## Discussion

Both theoretical and experimental studies suggest^6^,^8^,^18^,^19^,^20^,^21^ that *OM*_*ss*_ in sea spray is driven by insoluble gel-colloids forming material characterized by surface-active properties, and nanosized particles (<200 nm), which are components of *DOM*, such as viruses and cellular fragments due to cell death or cell burst induced by viral infection. This means that self-aggregation, forming insoluble surfactants, is the key process driving *OM*_*ss*_ in sea spray. *DOM* and *POM* in the ocean are in a dynamic equilibrium as the enrichment in DOM induces aggregation processes leading to the production of *POM*, while particle dissolution by extracellular enzymatic activities determines the shift from *POM* to *DOM*^22^. The aggregation properties of marine *OM* can be due to a number of factors, including presence of metals, dynamic conditions of the oceans, *UV* irradiation and chemical composition^23^. Moreover, the role of prokaryotic activity and cell lysis in forming such colloidal gel-forming material is proposed in several studies^24^,^25^,^26^. Indeed, our bubble bursting experiments support the above suggestions through revealing enrichment factors in fine mode aerosol of the order of 200 for viruses, 30 for prokrayotes, 100,000 for lipids, carbohydrates and proteins, and 15,000 for DNA. The enrichment factor in the fine, or submicron sea-spray was typically more than 10-fold that in the coarse mode.

While these processes are poorly understood at present, increasing evidence indicates that biological processes determining cell lysis, including viral infections could play a pivotal role in the process of transformation of living biomass into cell debris and in *DOM* dynamics (e.g. cytoplasmic material)^17^,^27^,^28^,^29^,^30^. Among biological processes responsible for the release of cell debris and *DOM* from phytoplankton blooms, grazing and viral infections can play a dominant role^30^,^31^. Marine phytoplankton blooms generally last for a few weeks and these biological processes can be responsible for the abatement of 50–100% of the phytoplankton. Zooplankton during grazing on phytoplankton, by sloppy feeding, can release approximately 30% of the ingested biomass as *DOM*.

Viruses play an important role in both global and small-scale biogeochemical cycling, influencing community structure and phytoplankton bloom termination. Viruses are indeed the most numerous ‘lifeforms’ in aquatic systems, at least 10 times the total number of Bacteria and Archaea, and an abundance in marine waters ranging from about 10^8^ to 10^10^ L^−1^. Given that the vast majority of the biomass in oceans comprises microorganisms, it is expected that viruses and other prokaryotic and eukaryotic microbes will play important roles in biogeochemical processes.

Viral infections in marine plankton can be lysogenic (viral genomes integrated into the genomes of their hosts) or lytic (causing host cell bursting), frequently followed by host death. Thus, lytic infections represent an important source of mortality of marine plankton.

Most marine viruses infect primarily prokaryotes, followed by microalgae, which are among the most abundant eukaryotic organisms in the ocean^27^,^32^. Virus-mediated mortality of bacterioplankton and phytoplankton is often in the range of 10–30%, but can reach 100%^33^,^34^. Epidemiological models predict that viral infection rates increase with increasing host cell density because infection is a direct function of the encounter rate between a pathogen and its host, and it is thought that host density drives much of the lytic response. For this reason, available studies provided evidence that viral abundance, and the importance of viruses in infecting and killing their hosts increases with the productivity of water masses, with the highest percentage of infected cells occurring in highly eutrophic ecosystems^35^. Phytoplankon bloom collapse occurs when viral lysis rates exceed the specific host growth rates. This type of interaction has been referred to as control by “reduction^30^”. Compelling evidence that viruses are involved in algal bloom demise comes from observations of high proportions (10–50%) of cells being visibly infected (using TEM) at the end of *Aureococcus anophagefferens*^36^, *Heterosigma akashiwo*^37^, and *Emiliania huxleyi*^38^ blooms. Further evidence for viral control of phytoplankton comes from dividing empirical estimates of virus production by burst size, which indicates that viral lysis can be responsible for 7 to 100% of the mortality of *Phaeocystis globosa*^39^ and *E. huxleyi*^40^ during the bloom events. When viral infections of phytoplankton provoke bursting of infected cells, their cellular content is released^30^,^41^, resulting in sub-micron sized particles and colloids (e.g., cell wall fragments and intracellular organic compounds) being released into surface waters^27^,^33^. Phytoplankton blooms are also known to produce exopolymeric substances^30^, which add to the variable and diverse *OM* pool, as a response to stress.

Wind-driven dynamics, resulting in bubble generation, is perhaps the most effective way of promoting self-aggregation and the formation of ocean surface foam-surfactant layers leading to *OM*_*ss*_. The significant difference between our results and those reporting low and invariant enrichment controlled by the *DOC* reservoir^3^ could be due to lack of wind-driven dynamics in those experiments. The difference could also be, in part, species driven: in the blooming northeast Atlantic waters, characterized by lower *Chl-a*, and similar *POC* and *DOC* concentrations (1.4 ± 0.8 μg L^−1^, 12 ± 6 μM C, 54 ± 20 μM C, respectively) compared to the northwest Atlantic and Pacific waters , *OM*_*ss*_, variable and far greater than 35% was encountered, pointing to the possibility that the very low and constant enrichment observed^3^ in regions of very high *Chl-a* concentration could be due to the production of *OM* which is lacking in aggregating surface-active properties. Such a scenario could arise, for instance, as a result of a lack of significant viral infections and the subsequent release of colloidal material and cell debris.

The increased correlation between *OM*_*ss*_ and biological activity indicators, as the timescale is increased, supports the concept that the production of *OM*, and its mass fraction enrichment in spray is, indeed, strongly correlated to the degree of surface water biological activity; however, the production of *OM* appears to be more linked to the bloom’s decay and cell lysis rather that life cycle – the waning of prokaryotes (bacteria and archaea) and phytoplankton due to viral infections after the waxing stage. In particular, these results are consistent with the time lag between the initiation of the phytoplankton bloom (generally assumed to occur when *Chl-a* concentrations pass the threshold of 1 μg L^−1^), the increase of prokaryotes that can exploit the phytoplankton exudates and the ability of viruses to infect living cells^33^,^42^. These aspects could contribute to explain why the phytoplankton biomass (here measured through the proxy *Chl-a* concentration) in surface waters is a pre-requisite for the release of sub-micron organic particles, mainly water insoluble, in the aerosol, but are not sufficient to determine the process directly. Aggregated biogenic materials are evident in both sea spray and filtered water images; however, it is predominantly fine (<10 μm) *POC* that contributes to sea spray *OM*_*ss*_ as evidenced by ^1^H-Nuclear Magnetic Resonance (^*1*^*HNMR*) fingerprinting of both fine *POC* and *OM* samples^8^. Colloids and nanogels, which are sufficiently small to enter the *DOC* operational definition (organic matter small enough in size to pass through a 0.2 μm pore filter) contribute to sea spray enrichment as well. We hypothesise that the viruses, which have been reported to increase exponentially along with the waning of *E. huxleyi* in the experimental systems show a size ranging from 120 to 180 nm and contribute to the *DOC* pool thought they are evidently submicron particles while the rest of the dissolved *OM* pool is less likely to contribute to enrichment in sea spray aerosol. The identification of the biological processes involved in the termination of the phytoplankton blooms, including the impact of viruses, is crucial to quantify their role in *DOM* dynamics. Further investigations are needed to provide mechanistic evidence of the interactions amongst these biotic components, able to provide production “pulses” of living and dead biomass. The bloom demise, or its death disco, not only forms an essential component of the climate-mediated plankton dance^43^, but also plays an essential role in the organic carbon cycle.

## Methods

### Aerosol Measurements

Aerosol chemical composition was measured with the Aerodyne Research Inc. high resolution time-of-flight aerosol mass spectrometer (*AMS*) which provides real-time size resolved composition analysis of volatile and semi-volatile particulate matter^12^. The combination of size and chemical analysis of *PM* 1.0 aerosol mass loading with fast time resolution makes the *AMS* unique and the theory of operation is well established. In summary, the *AMS* quantifies non-refractory aerosol chemical composition, covering major inorganic species such as ammonium, sulphate, nitrate, plus organic species^13^. In addition, recent work has demonstrated that both sea salt mass and primary marine organic mass can be retrieved from the high resolution *AMS*^14^. *GF* properties of aerosol were measured using a Hygroscopic Tandem Differential Mobility Analyser (*H-TDMA*)^44^. A higher *GF* indicates more hygroscopic particles resulting from a higher affinity for water. The Mace Head *H-TDMA* covered a “dry-size” *RH* of 20% to a humidified *RH* of 90%. GFs were measured for particle sizes of 35, 50, 75, 110 and 165 nm. An inversion algorithm was used to retrieve the *GF* Probability Density Function (*PDF*), adjusted for multiple charging effects and other required associated corrections^45^.

### Bubble-mediated experiments

A series of bubble-mediated aerosol production experiments were carried out using different cell densities and different stages of the of the microalgal species *Emiliania huxleyi* growth phase was examined. The cell densities chosen were typical of the abundance associated with natural bloom conditions. During each experiment, the cell number and viability was tested periodically; cell number was noted at the start of the experiment using a haemocytometer. The numbers of dead cells were counted during or at the end of the experiment by adding 2–3 drops of 0.1% Evan’s blue dye to 20 mL of the sample^46^. Samples were also collected for *Chl-a* (HPLC), nutrient and *DOC* and *POC* analysis. *WSOM* and *WIOM* for selected experiments were also analysed by ^*1*^*HNMR* analysis.

In the experiments, an airtight high grade stainless steel tank (200 L in volume) was filled with 100 L of filtered and *UV*-sterilised seawater. This seawater was freshly collected close to Mace Head in Carna Bay, Co. Galway, on the day each experiment. Samples were taken from approximately 30 m off-shore. Light was provided by cool white fluorescent lamps (L 18W/865 lumilux fluorescence lamps, Osram, Germany) fitted above the water surface inside the tank which provided the phytoplankton cultures a constant light of 80 μmol photons m^−2^ s^−1^ (measured using a Li Cor 1400 data logger) at 12 h:12 h photoperiod. Air was entrained into water from the top of the tank by a vertical re-circulating water jet at a flow rate of 20 L min^−1^. The entrained air formed bubbles that in turn burst at the surface producing sea spray aerosol.

The tank had several ports on the top where different analytical instruments were connected: (1) an *AMS*, which measured sea spray chemical composition; (2) two 8 stage Berner impactors mounted for approximately 6 hours (0.060–16 μm diameter), with different sampling substrates (quartz and tedlar) in order to analyse the organic and inorganic components; (3) a Scanning Mobility Particle Sizer (SMPS), which measured aerosol size distributions (10–500 nm); (4) a Humidified Tandem Differential Mobility Analyser (*HTDMA*), which measured aerosol growth factor (35, 50, 75, 110 and 165 nm).

Each experiment was carried out for up to several days until after the phytoplankton culture was declared dead. Two experiments were specifically designed to study algae-virus interaction using *Emiliania huxleyi* (CCMP 1516) and cocolithovirus EhV-86 with corresponding densities of 10^5^ cells ml^−1^ and 10^4^ viruses ml^−1^ which were cultured in separate batches and inoculated 4 hours before being transferred into experimental tank. A separate control tank was used in algae-virus experiments using same phytoplankton culture and density as in experimental tank, but without inoculation with viruses.

### Description of the Satellite Products

Daily satellite sea surface *Chl-a* concentration data were acquired from MyOcean^47^. The global *Chl-a* dataset at 4 km of resolution produced by the ESA OC-CCI Project was used and is a climate-quality consistent dataset produced using the latest and most complete knowledge of satellite sensor calibration, characterization and attitude, complete ancillary data sets, latest versions of models and algorithms. SeaWiFS, MODIS, and MERIS Remote sensing reflectances were corrected for atmospheric transmission, band shifted to SeaWiFS wavebands, and merged. *Chl-a* concentration were calculated applying the NASA OC4.V6 algorithm on the OC-CCI merged remote sensing reflectances^48^. Standard masking criteria for detecting clouds or other contamination factors were applied to the sensor data, i.e., land, cloud, sun glint, atmospheric correction failure, high total radiance, large solar zenith angle (70°), large spacecraft zenith angle (56°), coccolithophores, negative water leaving radiance, and normalized water leaving radiance at 555 nm 0.15 Wm^−2^ sr^−1^ 49. The quality of chlorophyll product (mg m^−3^) was evaluated by comparing the satellite retrieval with corresponding *in situ* data. The analysis, performed on the log_10_ transformed data, results in a bias of −0.01910 and an RMS 0.30300 (see MyOcean Quality Information Document: MYO2-OC-QUID-009–056–057–058–059-V2.0^50^).

From this global dataset we extracted the chlorophyll field our study area (29°-7°W; 44°-60°N) from 1^st^ January 2009 to 31^st^ December 2011. Multi-channel singular spectral analysis (M-SSA) was used to fill daily gaps in the chlorophyll maps due to cloud cover or other environmental factors. The method uses temporal as well as spatial correlation to fill the missing points (see Appendix A of Rinaldi *et al.*^10^ for method details).

The *NPP* dataset used in this paper were acquired from the Ocean Productivity webpage^51^. We selected the PP Standard product computed using the Vertically Generalized Production Model (VGPM)^52^. For the VGPM, net primary production is a function of chlorophyll, available light, and the photosynthetic efficiency. This Standard Product is computed using MODIS chlorophyll and temperature data, SeaWiFS PAR, and estimates of euphotic zone depth from a model developed by Morel and Berthon^53^ and based on chlorophyll concentration.

### Analyses of viruses and prokaryotes in the seawater and marine aerosol

Seawater, samples were filtered onto Anodisc 25 mm (20 nm pore size) filters. To determine viral and prokaryotic abundance in the aerosol, each size class quartz filter (collected for approximately 3–12 h during bubble bursting experiments by using an 80 L min^−1^ five stage Berner impactor in the size range 0.05–10 μm) was homogenised in sterile and virus free MilliQ water (pre-filtered onto 0.02 μm pore-size filters) and samples were then treated by ultrasounds and pyrophosphate (5 mM final concentration) to detach viruses and prokaryotes. Aerosol samples were then centrifuged at 800 *g* for 10 minutes and the supernatant filtered onto Anodisc 25 mm (20 nm pore size) filters. All Anodisc filters were then stained with 20 μl of SYBR Green I (diluted 20 fold in MilliQ water, optical density at 495 nm ca. 1.3) for 15 minutes in the dark, rinsed twice with 1 ml MilliQ water (in order to eliminate fluorescence background noise). The filters were mounted on slides using an anti-fade solution (50% phosphate buffer [6.7 mM, pH 7.8] and 50% glycerol containing 0.25% ascorbic acid) and analyzed by epifluorescence microscopy using a Zeiss Axioplan microscope, equipped with a 50W lamp. To reduce the uncertainties on viral and prokaryotic counts due to the low abundance in aerosol samples, at least 100 optical fields at 1000× magnification were examined.

### Analyses of carbohydrates, proteins, lipids and DNA in the seawater and marine aerosol

Seawater samples were pre-filtered onto 10 μm pore-size filters and then onto polycarbonate 0.2 μm pore-size filters. Each size class quartz filter used for collecting aerosol during bubble bursting experiments was homogenised in sterile and virus free MilliQ water (pre-filtered onto 0.02 μm pore-size filters) and samples were then treated with ultrasound. Carbohydrate, protein and lipid determinations, both in aerosol and seawater samples, were carried out spectrophotometrically according to procedures previously described^54^. The analyses of total DNA in aerosol and seawater samples were performed fluorometrically using SYBR Green I as fluorochrome after digestion of RNA with RNase (DNase free, 1 U mL^−1^ for 15 minutes at room temperature). Carbohydrate, protein, lipid and DNA concentrations were obtained by calibration curves using standard solutions of glucose (1–25 μg mL^−1^), bovine serum albumin (0.5–10 μg mL^−1^), tripalmitin (2.5–25 μg mL^−1^) and DNA from *E. coli* (1–100 ng mL^−1^), respectively. The detection limits were 2.5 μg mL^−1^, 1 μg mL^−1^, 5 μg mL^−1^ and 2.5 ng mL^−1^ for carbohydrates, proteins, lipids and DNA, respectively. Clean quartz and polycarbonate filters were used as blanks. Carbohydrate, protein, lipid and DNA concentrations obtained from the analyses of 0.05–1.2 μm pore size filters were summed and defined as belonging to the fine fraction (<1.2 μm) of the aerosol, whereas concentrations obtained from the analyses of 1.2–10 μm pore size filters were defined as belonging to the coarse fraction (>1.2 μm).

## Additional Information

**How to cite this article**: O’Dowd, C. *et al.* Connecting marine productivity to sea-spray *via* nanoscale biological processes: Phytoplankton Dance or Death Disco? *Sci. Rep.* **5**, 14883; doi: 10.1038/srep14883 (2015).
